# Portal hypertension in prolonged anorexia nervosa with laxative abuse: A case report of three patients

**DOI:** 10.1002/eat.23007

**Published:** 2019-01-12

**Authors:** Aiko Koga, Kenta Toda, Keita Tatsushima, Sunao Matsuubayashi, Naho Tamura, Masatoshi Imamura, Keisuke Kawai

**Affiliations:** ^1^ Department of Psychosomatic Medicine Kohnodai Hospital, National Center for Global Health Medicine Ichikawa City Chiba Japan; ^2^ Department of Psychosomatic Medicine Fukuoka Tokusyuukai Hospital Kasuga City Fukuoka Japan; ^3^ Department of Gastroenterology and Hepatology Kohnodai Hospital, National Center for Global Health Medicine Ichikawa City Chiba Japan

**Keywords:** anorexia nervosa, case reports, eating disorders, laxatives, liver diseases, liver fibrosis, portal hypertension

## Abstract

**Objective:**

There has been no report on portal hypertension related to anorexia nervosa (AN).

**Method:**

We describe three cases of portal hypertension manifesting with collateral circulation represented by gastroesophageal varices in prolonged AN with laxative abuse and self‐vomiting. These women, in their 20s to 50s, were diagnosed as having AN binging and purging type (AN‐BP) that included self‐induced vomiting and abuse of irritating laxatives (more than 100 tablets daily).

**Results:**

Case 1 showed prominent ascites and a gastro‐renal shunt on computed tomography scanning. Case 2 showed gastroesophageal varices on endoscopic examination. Case 3 showed gastroesophageal varices on computed tomography scanning and endoscopic examination. We performed liver biopsies in all patients and found only slight pericellular fibrosis. Our patients showed typical symptoms of portal hypertension, although liver cirrhosis was not present.

**Discussion:**

We speculated that abnormal eating and purging behaviors were involved in the development of portal hypertension. We hypothesized that long‐term laxative abuse, dehydration, and abnormal eating behavior are involved in the development of portal hypertension, considering these were common features in our patients. Portal hypertension and gastroesophageal varices should be considered as one of the potentially existing complications in prolonged AN‐BP with self‐induced vomiting and abuse of irritating laxatives.

## 1 BACKGROUND

Portal hypertension is a severe complication of chronic liver disease represented by cirrhosis (Bosch, Abraldes, Berzigotti, & García‐Pagan, 2009). When the portal vein is under pressure, ascites can develop along with collateral circulations, represented by gastroesophageal varices, epigastric vein dilatation, and hemorrhage (Bosch et al., 2009; Bosch, Berzigotti, Garcia‐Pagan, & Abraldes, 2008). Anorexia nervosa (AN) sometimes manifests with severe malnutrition and organ damage. Different physical complications of AN can occur (Papadopoulos, Ekbom, Brandt, & Ekselius, 2009; Steinhausen, 2002; Treasure, Claudino, & Zucker, 2010). Autophagy, for example, in acute liver damage in cases of AN, has been reported (Rautou et al., 2008). There is no report on chronic liver alteration and portal hypertension related to AN. We describe three cases of portal hypertension in prolonged AN.

## 2 CASE PRESENTATIONS

The summary of each patient's background characteristics, clinical findings on admission, and psychological examination results on the first visit are shown in Table 1. A diagnosis of AN binging and purging type (AN‐BP) was based on the eating disorders section of the Structured Clinical Interview for DSM‐IV Axis I Disorders.

**Table 1:** Summary of each patient's background characteristics and clinical findings

	Case 1^b^	Case 2^b^	Case 3^b^
Background characteristics			
Age (years)/sex/diagnosis	52/female/AN‐BP	38/female/AN‐BP	29/female/AN‐BP
BMI (kg/m^2^)/disease duration (years)	15.4/20	15.8/15	14.8/10
Past history/alcohol habits	Endometriosis/–	Osteoporosis/–	Varices rupture/–
Abuse of OCT^c^ laxatives, duration (years)/amount (tablets/day)	20/≥200	1/≥100	10/≥200
Laboratory values on admission			
Alb (g/dL)**/**Ch‐E (mg/dL)/T‐Chol (mg/dL)	1.8^a^/317/228.6	2.7^a^/206/237	2.5^a^/206/124^a^
AST (U/L)/ALT (U/L)	79^a^/62^a^	70^a^/59^a^	41^a^/34^a^
ALP (U/L)/γ‐GTP (U/L)/T‐Bil (mg/dl)	591^a^/115^a^/0.5	1,747^a^/467^a^/0.2	320/45^a^/0.2
BUN (mg/dL)/Cre (mg/dL)	12/1.62^a^	11.7/1.01^a^	178^a^/2.47^a^
Na (mmol/L)/K (mmol/L)	136^a^/3.2^a^	138/4	121^a^/5.2^a^
RBC count (/μL)/Hb (g/dL)	3,170,000^a^/10.2^a^	3,190,000^a^/9.3^a^	2,710,000^a^/8.3
WBC (/μL)/TLC (/μL)	8,500/1,700	10,330/1,859	8,400/1,400
Plt count (/μL)	324,000	320,000	269,000
PT (%)s	81.6	131	81.7
Physical manifestations			
Collateral circulation	Gastro‐renal shunt	Esophageal varices	Gastric varices
	Epigastric vein dilatation		Esophageal varices
	Striae cutis distensae		Gastro‐renal shunt
Body fluid distribution	Ascites	Systemic edema	Ascites
Psychological examination			
EDI‐2/EAT26	Not done/54	Not done	65/15
STAI/CESD/TAS20	S‐70 T‐55/36/25	Not done	S‐54 T‐66/31/55

*Note*. Alb = albumin (4.1–5.1 g/dL); ALP = alkaline phosphatase (106–322 U/L); ALT = alanine aminotransferase (7–23 U/L); AN‐BP = anorexia nervosa binge‐purge type; AST = aspartate aminotransferase (13–30 U/L); BMI = body mass index; BUN = blood urea nitrogen (8–20 mg/dL); CESD = The Center for Epidemiologic Studies Depression Scale; Ch‐E = cholinesterase (201–421 U/L); Cre = creatinine (0.46–0.79 mg/dL); EAT26 = Eating Attitudes Test‐26; EDI‐2 = Eating Disorder Inventory‐2; Hb = hemoglobin (11.6–14.8 g/dL); K = potassium (3.6–4.8 mmol/L); Na = sodium (138–145 mmol/L); OCT = Over the counter; Plt = platelet (15,800–34,800/μL); PT = prothrombin time (70–140%); RBC = red blood cell (386–492/μL); S‐ = state score; STAI = State–Trait Anxiety Inventory; T‐ = trait score; TAS20 = The 20‐Item Toronto Alexithymia Scale; T‐Bil = total bilirubin (0.4–1.5 g/dL); T‐Chol = total cholesterol (142–248 mg/dL); TLC = total lymphocyte count; WBC = white blood cell (3,300–8,600/μL); γ‐GTP = gamma‐glutamyl transpeptidase (9–32 U/L). ( ) Normal range.

a Abnormal range.

b Cases 1 and 3 were from Khonodai Hospital; Case 2 was from Fukuoka Tokusyukai Hospital.

### 2.1 Case 1

**Figure 1:**
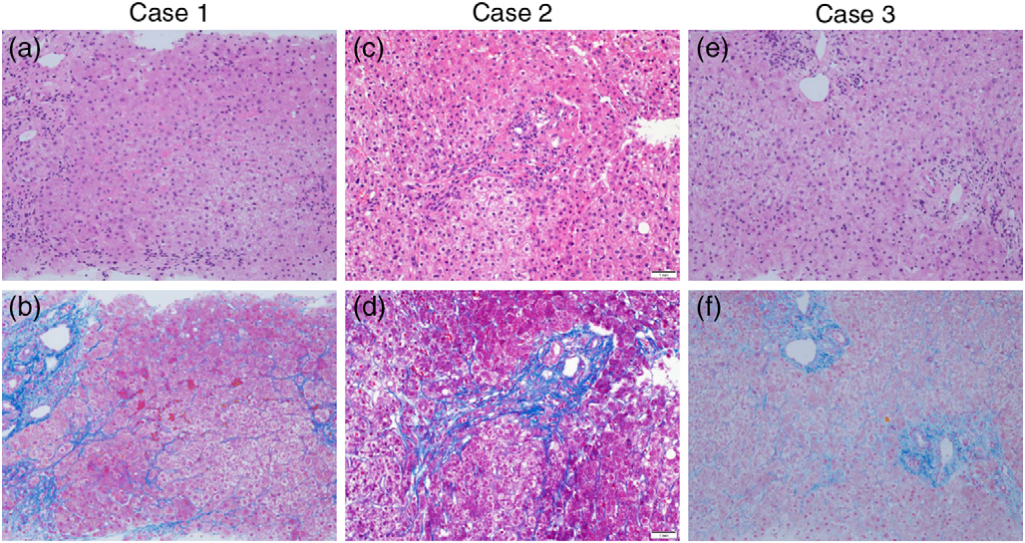
Histopathological findings. Hematoxylin and eosin staining of liver biopsy specimens of Case 1 (a), Case 2 (c), and Case 3 (e) (original magnification ×20). Azan staining of liver biopsy specimens of Case 1 (b) and Case 3 (f) (original magnification ×20). Mallory–Azan staining of liver biopsy specimens of Case 2 (d) (original magnification ×20). All patients show pericellular fibrosis but no evidence of liver cirrhosis

This patient was a 52‐year‐old woman with AN‐BP, who had been involved in self‐induced vomiting and laxative abuse of more than 200 tablets per day for 20 years. She had been administered supportive psychotherapy and symptomatic treatment by psychiatrists including hospitalization at 13 instances. She visited us for evaluation of prominent ascites and treatment of AN‐BP. On physical examination, epigastric vein dilatation and striae cutis distensae of the swelling abdomen, which was filled with ascites, were seen. Blood test results indicated hypoalbuminemia, increased hepatic and biliary enzyme levels, an increased creatinine level, hypokalemia, and anemia (Table 1). The abdominal ultrasonogram showed findings of chronic hepatitis: an irregular liver surface, a dull margin, and heterogeneity of the parenchyma. On the computed tomography scan, a gastro‐renal shunt was found, although no varices were detected. Hence, portal hypertension due to liver cirrhosis was suspected. Therefore, we performed a liver biopsy. However, only slight pericellular fibrosis was observed without evidence of liver cirrhosis (Figure 1a,b). Liver fibrosis was also proven by the increase in liver fibrosis makers of type IV collagen 7S (8.5 ng/mL) and hyaluronic acid (400 ng/mL). On psychological testing, depression and anxiety were remarkable (Table 1). During inpatient care, we provided nutritional treatment and diuretic drugs to control ascites. After her general state improved, an inpatient cognitive behavioral treatment was initiated. She was discharged 2 months after admission because the ascites almost disappeared and her body weight increased by approximately 3 kg. However, serum levels of aspartate aminotransferase (AST), alanine aminotransferase (ALT), alkaline phosphatase (ALP), gamma‐glutamyl transpeptidase (γ‐GTP), and creatinine were not significantly improved. During our 3‐month follow‐up, her ascites were well controlled with diuretic drugs, and we instructed her to limit her daily activities. As for the aforementioned serum levels, no changes were observed.

### 2.2 Case 2

This patient was a 38‐year‐old woman with AN‐BP for 15 years. In the first 14 years, her purging behavior only included binging and self‐induced vomiting. Laxative abuse started over the last year, and she consumed 100 tablets or more daily. Before our intervention, she had been hospitalized repeatedly by physicians and surgeons for infusion therapy in order to improve dehydration and electrolyte imbalance. She was admitted to our department because of severe malaise, systemic edema, and liver malfunction. High levels of ALP and γ‐GTP were remarkable on the blood test (Table 1). ALP‐2, an ALP isozyme derived from the liver was remarkably raised to 72%. Hypoalbuminemia, increased AST and ALT levels, high creatinine level, and anemia were also indicated. An abdominal ultrasonogram confirmed ascites and a chronic hepatitis pattern. On endoscopic examination, we observed esophageal varices, the gross appearance of which was categorized as located on the lower third of the esophagus (Li), straight form (F1), blue color (Cb), and slight red cherry sign (RC1), according to the Japanese Research Society for Portal Hypertension (JRSPH) classification. No abnormalities were detected on computed tomography. Liver biopsy results revealed slight pericellular fibrosis, but neither bridging fibrosis nor liver cirrhosis (Figure 1c,d). The increase in liver fibrosis markers M2BPGi (1.34 COI), type IV collagen 7S (530 ng/mL), and hyaluronic acid (721 mg/dL) was observed. As for inpatient care, diuretic drugs (spironolactone, 25 mg/day and tolvaptan, 7.5 mg/day) were administered in order to control systemic edema and ascites. After 2 weeks of hospitalization, she was discharged with decreased edema and ascites, but there was no change in AST, ALT, ALP, and γ‐GTP levels. Four months later, neither the recurrence of edema and ascites nor the improvement in hepatic enzyme level was seen. Coexistence of a borderline personality disorder complicated her treatment. We performed psychotherapy based on motivational interviews.

### 2.3 Case 3

This patient was a 29‐year‐old woman with AN‐BP, who had been involved in self‐induced vomiting and laxative abuse of more than 200 tablets per day for approximately 10 years. We provided her with supportive psychotherapy and psychoeducation at our outpatient clinic, along with impatient care as needed. Anxiety, depression, and difficulties in self‐identity and expression were distinctive on her psychological test (Table 1). Her condition was also complicated with chronic kidney disease; her serum creatinine level was generally 1.5–2.0 mg/dL. She had a medical history of ruptured esophageal varices, for which endoscopic variceal ligation was conducted. The varices ruptured again, and inpatient care was given in the Gastrointestinal Department. On physical examination, epigastric vein dilatation and hemorrhage were observed. The blood test results on admission are presented in Table 1. The enhanced computed tomography scan revealed gastroesophageal varices and a gastro‐renal shunt. The endoscopic examination showed gastric varices, classified as gastro‐renal shunt accompanied with gastric varices (Lg‐f), enlarged and tortuous (F2), white color (Cw), and no red color sign (RC0), and esophageal varices categorized as Li, F1, Cb, and RC1 according to the JRSPH classification. On liver biopsy, there was only slight pericellular fibrosis and again no finding of liver cirrhosis (Figure 1e,f). A high level of M2BPGi (1.56 COI) was also confirmed. On admission, 4 U of red blood cells were infused, and endoscopic variceal ligation was performed. Her hemoglobin level recovered from 5.6 to 10.4 mg/dL, and hemostasis was confirmed on second‐look endoscopy. The serum creatinine level decreased from 2.47 to 1.02 mg/dL with hydration. Hyperkalemia also improved in accordance with the recovery of renal function, and consequently, returned to hypokalemia of 2–3 mmoL/L, which was the usual level for her. Psychosomatic doctors provided supportive psychological interviews, and her AN‐BP state remained stable. She was discharged after 11 days of hospitalization. Subsequently, she underwent balloon‐occluded transfemoral obliteration for the gastric varices. Eleven months later, dissipation of the gastric and esophageal varices was revealed on endoscopic follow‐up.

## 3 DISCUSSION

Clinically significant portal hypertension is defined as an increase in hepatic venous pressure gradient (HVPG) to ≥10 mmHg (Bosch et al., 2009). Hypertension in the portal vein occurs when there is resistance to portal blood flow or an increase in the amount of blood flowing into the portal system (Bosch et al., 2009). Cirrhosis is the main cause of portal hypertension. Thrombosis and stenosis at the portal vein or hepatic vein also increase resistance to the blood stream (Bosch et al., 2008). Primary sclerosing cholangitis and primary biliary cirrhosis (LaRusso, Wiesner, Ludwig, & MacCarty, 1984; Lazaridis & Gores, 2006), and drug‐induced liver damage can result in portal hypertension (Bosch et al., 2008).

AN is a disease caused by an intense fear of gaining weight and ends in a state of extreme weight loss, despite no basal organic disease. There are various physical complications due to malnutrition and purging behavior (Westmoreland, Krantz, & Mehler, 2016). Renal failure, for example, is caused by dehydration and hypokalemia owing to self‐induced vomiting and use of laxatives or diuretic drugs in addition to an insufficient intake of water (Takakura et al., 2006; Westmoreland et al., 2016). Liver transaminase levels elevate to different extents. AST and ALT are the most frequently affected levels in a range of 2–4 times the normal limit, but sometimes they increase to higher levels (Narayanan, Gaudiani, Harris, & Mehler, 2010; Norris et al., 2016; Rosen et al., 2016; Westmoreland et al., 2016). Even sudden death due to cardiac disorders, such as arrhythmia can occur (Westmoreland et al., 2016).

Among our patients, the following clinical and pathological features were found: (a) prolonged duration of disease, (b) habitual self‐induced vomiting, (c) abuse of irritating laxatives, and (d) histological findings of hepatic inflammatory reaction but no cirrhosis on liver biopsy. As a supplement, the laxatives that the three patients abused were commercially available ones, and mainly consisted of bisacodyl and sennosides. However, these laxatives were not necessarily a particular product nor produced by a single manufacturer.

In addition, several intriguing points were noted. First, neither pedal edema nor plural effusion was seen in Cases 1 and 3 (Table 1), although ascites was detected by ultrasonography and computed tomography. This suggests that hypoalbuminemia was not the only cause of exudation, and hydrostatic pressure limited to intraperitoneal circulation must have been involved. This disproportion of localized effusion may have contributed to the existence of portal hypertension. Moreover, systemic edema and the increase in ALP in Case 2 were not attributed to congestive liver subsequent to congestive heart failure based on her chest x‐ray examination, which did not show corresponding findings, such as cardiac dilatation and lung edema.

Second, we succeeded in proving hepatic fibrosis based on liver biopsy findings in our patients. According to an experiment that used rats, abundant consumption of irritating laxatives can induce liver fibrosis (Ma, Zheng, He, & Li, 2015). We found a notable description in *Disease of the liver and biliary* (Eleventh ed.) by Sherlock and Dooley (2008), of an animal experiment that revealed that liver fibrosis can be induced by malnutrition. Additionally, fibrosis of cardiac muscles (Oflaz et al., 2013) and fibrosis with acute sever liver damage in AN (Rautou et al., 2008) have already been reported. However, fibrosis accompanied with chronic liver damage in AN has never been focused on until our report.

Third, it was surprising that portal hypertension in our patients was not preceded by liver cirrhosis. Generally, fibrosis occurs over the course of an inflammatory reaction, and highly developed fibrosis results in cirrhosis (Friedman, 1993), causing portal hypertension. Our patients' liver biopsy results only revealed slight pericellular fibrosis.

Regarding differential diagnosis of chronic liver damage and portal hypertension in our cases, viral hepatitis, primary sclerosing cholangitis, and primary biliary cholangitis (Bosch et al., 2008) were ruled out based on laboratory data and histopathological findings (Dhiman et al., 2002; Lee & Kaplan, 1995; Scheuer, 1998). Idiopathic portal hypertension was unlikely, as the typical features of splenomegaly and portal fibrosis (Dhiman et al., 2002) were not observed. Although metabolic impairments such as hemochromatosis, alpha‐1 antitrypsin deficiency, and porphyria are known as causes of chronic liver disease (Heidelbaugh & Bruderly, 2006), there were no relevant clinical findings and history indicating these diseases. Neither alcohol habits nor pharmacological history of high hepatotoxicity, such as methotrexate and isoniazids (Heidelbaugh & Bruderly, 2006) was seen. Additionally, somnifacients, antianxiety agents, and a ferric medicine, for instance, were prescribed depending on each patient's condition, within the normal dose. There was no common medicine that all three patients were taking.

We describe our pathological hypothesis concerning this issue. We speculate that three distinctive features of AN‐BP, malnutrition, laxative abuse, and self‐induced vomiting, would affect the development of portal hypertension majorly and reciprocally. Hypoalbuminemia due to malnutrition, laxative use, and emesis cause endovascular dehydration. Moreover, hypoperfusion due to the dehydration causes renal dysfunction and hepatic disorder (Ma et al., 2015; Takakura et al., 2006). Malnutrition also leads to liver damage (Narayanan et al., 2010). Moreover, renal parenchymal failure owing to hypokalemia based on insufficient intake of potassium, laxative use, and emesis occurs concomitantly with prerenal failure because of dehydration (Takakura et al., 2006). We predict that these renal and hepatic malfunctions cause some hemodynamic changes, which result in the development of portal hypertension. Additionally, hypoperfusion might induce peripheral portal vein collapse and can increase blood flow resistance of the portal system.

We suggest that a patient with clinical features corresponding to clinical and pathological features 1–3 that we proposed should be suspected of having portal hypertension. This is because the advanced therapeutic strategy can be based on ascites and gastroesophageal varix derived from portal hypertension. Persistent appetite loss would be associated with abdominal discomfort and less bowel movements caused by ascites. In such a case, control of ascites is required. Furthermore, nasoenteric feeding should be applied carefully; otherwise, a latently existing varix on the upper digestive tract could become ruptured through the invasive process of inserting a nasogastric tube. This article also indicates that the potential hepatotoxicity of irritating laxatives should be recognized in our daily clinical practice. Yet, further studies should be conducted so that portal hypertension can be established as a complication of AN.

There are a few limitations of our study. First, the current report includes a small sample size. Second, thrombosis and stenosis of the portal or hepatic vein (Bosch et al., 2008) were not completely excluded unless coagulation state and enhanced computed tomography were obtained. Third, the measurement of free portal vein pressure and wedged hepatic vein pressure, as well as HVPG (Bosch et al., 2009) would be crucial to reveal the pathology.

## 4 CONCLUSIONS

In this research, three clinical cases were presented, in which portal hypertension and accompanied gastroesophageal varices occurred in patients with a long history of AN, habitual self‐induced vomiting, abuse of irritating laxatives, and hepatic failure. We predict that the interaction of multiple factors of AN results in hemodynamic changes. However, a univocal hypothesis cannot be formulated, especially in light of the small sample size, lack of information such as coagulation state, enhanced computed tomography, HVPG, and so forth.

## INFORMED CONSENT

Informed consent was obtained in writing for publication of this case report and accompanying images from each patient.

## References

[eat23007-bib-0001] Bosch, J. , Abraldes, J. G. , Berzigotti, A. , & García‐Pagan, J. C. (2009). The clinical use of HVPG measurements in chronic liver disease. Nature Reviews Gastroenterology & Hepatology, 6, 573–582. 10.1038/nrgastro.2009.149 19724251

[eat23007-bib-0002] Bosch, J. , Berzigotti, A. , Garcia‐Pagan, J. C. , & Abraldes, J. G. (2008). The management of portal hypertension: Rational basis, available treatments and future options. Journal of Hepatology, 48, S68–S92. 10.1016/j.jhep.2008.01.021 18304681

[eat23007-bib-0003] Dhiman, R. K. , Chawla, Y. , Vasishta, R. K. , Kakkar, N. , Dilawari, J. B. , Trehan, M. , … Suri, S. (2002). Non‐cirrhotic portal fibrosis (idiopathic portal hypertension): Experience with 151 patients and a review of the literature. Journal of Gastroenterology and Hepatology, 17(1), 6–16. 11895549 10.1046/j.1440-1746.2002.02596.x

[eat23007-bib-0004] Friedman, S. L. (1993). Seminars in medicine of the Beth Israel Hospital, Boston. The cellular basis of hepatic fibrosis: Mechanisms and treatment strategies. The New England Journal of Medicine, 328, 1828–1835. 10.1056/NEJM199306243282508 8502273

[eat23007-bib-0006] Heidelbaugh, J. J. , & Bruderly, M. (2006). Cirrhosis and chronic liver failure: Part I. Diagnosis and evaluation. American Family Physician, 74(5), 756–762. 16970019

[eat23007-bib-0007] LaRusso, N. F. , Wiesner, R. H. , Ludwig, J. , & MacCarty, R. L. (1984). Primary sclerosing cholangitis. The New England Journal of Medicine, 310, 899–903. 6366557 10.1056/NEJM198404053101407

[eat23007-bib-0008] Lazaridis, K. A. , & Gores, G. J. (2006). Primary sclerosing cholangitis and cholangiocarcinoma. Seminars in Liver Disease, 26, 42–51. 10.1016/S0140-6736(18)30300-3 16496232

[eat23007-bib-0009] Lee, Y. M. , & Kaplan, M. M. (1995). Primary sclerosing cholangitis. The New England Journal of Medicine, 332, 924–933. 10.1056/NEJM199504063321406 7877651

[eat23007-bib-0010] Ma, J. , Zheng, L. , He, Y. S. , & Li, H. J. (2015). Hepatotoxic assessment of polygoni multiflori radix extract and toxicokinetic study of stilbene glucoside and anthraquinones in rats. Journal of Ethnopharmacology, 162, 61–68. 10.1016/j.jep.2014.12.045 25557036

[eat23007-bib-0011] Narayanan, V. , Gaudiani, J. L. , Harris, R. H. , & Mehler, P. S. (2010). Liver function test abnormalities in anorexia nervosa—Cause or effect. The International Journal of Eating Disorders, 43(4), 378. 19424979 10.1002/eat.20690

[eat23007-bib-0012] Norris, M. L. , Harrison, M. E. , Isserlin, L. , Robinson, A. , Feder, S. , & Sampson, M. (2016). Gastrointestinal complications associated with anorexia nervosa: A systematic review. The International Journal of Eating Disorders, 49(3), 216–237. 10.1002/eat.22462 26407541

[eat23007-bib-0013] Oflaz, S. , Yucel, B. , Oz, F. , Sahin, D. , Ozturk, N. , Yaci, O. , … Oflaz, H. (2013). Assessment of myocardial damage by cardiac MRI in patients with anorexia nervosa. The International Journal of Eating Disorders, 46(8), 862–866. 10.1002/eat.22170 23922168

[eat23007-bib-0014] Papadopoulos, F. C. , Ekbom, A. , Brandt, L. , & Ekselius, L. (2009). Excess mortality, causes of death and prognostic factors in anorexia nervosa. The British Journal of Psychiatry: The Journal of Mental Science, 194(1), 10–17. 10.1192/bjp.bp.108.054742 19118319

[eat23007-bib-0015] Rautou, P. E. , Cazals‐Hatem, D. , Moreau, R. , Francoz, C. , Feldmann, G. , Lebrec, D. , … Durand, F. (2008). Acute liver cell damage in patients with anorexia nervosa: A possible role of starvation‐induced hepatocyte autophagy. Gastroenterology, 135(3), 840–848, 848.e1–3. 10.1053/j.gastro.2008.05.055 18644371

[eat23007-bib-0016] Rosen, E. , Sabel, A. L. , Brinton, J. T. , Catanach, B. , Gaudiani, J. L. , & Mehler, P. S. (2016). Liver dysfunction in patients with severe anorexia nervosa. The International Journal of Eating Disorders, 49(2), 151–158. 10.1002/eat.22436 26346046

[eat23007-bib-0017] Scheuer, P. J. (1998). Ludwig symposium on biliary disorders—Part II. Pathologic features and evolution of primary biliary cirrhosis and primary sclerosing cholangitis. Mayo Clinic Proceedings, 73(2), 179–183. 9473003 10.4065/73.2.179

[eat23007-bib-0018] Sherlock, S. , & Dooley, J. (2008). Disease of the liver and biliary (11th ed). Hoboken, NJ: Wiley‐Blackwell.

[eat23007-bib-0019] Steinhausen, H. C. (2002). The outcome of anorexia nervosa in the 20th century. The American Journal of Psychiatry, 159(8), 1284–1293. 10.1176/appi.ajp.159.8.1284 12153817

[eat23007-bib-0020] Takakura, S. , Nozaki, T. , Nomura, Y. , Koreeda, C. , Urabe, H. , Kawai, K. , … Kubo, C. (2006). Factors related to renal dysfunction in patients with anorexia nervosa. Eat and Weight Disorders, 11(2), 73–77. 10.1007/BF03327754 16809978

[eat23007-bib-0021] Treasure, J. , Claudino, A. M. , & Zucker, N. (2010). Eating disorders. Lancet, 375(9714), 583–593. 10.1016/S0140-6736(09)61748-7 19931176

[eat23007-bib-0022] Westmoreland, P. , Krantz, M. J. , & Mehler, P. S. (2016). Medical complications of anorexia nervosa and bulimia. The American Journal of Medicine, 129(1), 30–37. 10.1016/j.amjmed.2015.06.031 26169883

